# Osteocalcin and frailty among older women

**DOI:** 10.1007/s40520-025-03239-6

**Published:** 2025-12-09

**Authors:** Tine Kolenda Paulin, Linnea Malmgren, Patrik Bartosch, Kaisa K. Ivaska, Fiona E. A. McGuigan, Kristina E. Akesson

**Affiliations:** 1 Clinical and Molecular Osteoporosis Research Unit, Department of Clinical Sciences, Lund University, Malmö, Sweden; 2 Department of Geriatrics, Skåne University Hospital, Clinical Research Centre, Jan Waldenströms gata 35, 214 28 Malmö, Sweden; 3 Institute of Biomedicine, University of Turku, Turku, 20520 Finland; 4 Department of Orthopaedics, Skåne University Hospital, Malmö, Sweden

**Keywords:** Osteocalcin, Frailty, Bone turnover markers, Old individuals

## Abstract

**Background:**

Osteocalcin is a bone-specific protein involving many physiological processes, primarily bone turnover. Also closely related to the musculoskeletal system is the frailty syndrome.

**Aim:**

To investigate if circulating osteocalcin levels and frailty are associated in the old, and in addition, if the presumed association is mediated through alterations in bone.

**Methods:**

999 community-dwelling women from the OPRA (Osteoporosis Prospective Risk Assessment) cohort, all aged 75 years. Serum total osteocalcin was measured together with bone turnover markers PINP and CTX. An OPRA-adapted frailty index was applied. Association between osteocalcin and frailty was investigated using both logistic regression (osteocalcin quintiles Q_low_-Q_high_; Q_1_-Q_5_) and linear regression. Splines model was added. Association between osteocalcin level and individual components of the frailty index were investigated using Kruskal-Wallis or Chi^2^ test.

**Results:**

Low osteocalcin (Q_1_) was associated with being frail (frailty prevalence 36% vs. 23% (Q1 vs. Q5); absolute difference 13%) in both unadjusted (OR_unadj_ 1.82, 95% CI[1.12-3.00]) and adjusted analyses (OR_adj_ 2.55, 95% CI[1.46–4.44]); even after adjustment for bone turnover markers, s-PINP and s-CTX (2.50, 95% CI[1.11–5.61]). Women with low serum osteocalcin (Q1) had significantly poorer gait function (gait speed (*p* = 0.001; p for trend < 0.001), more steps taken (*p* = 0.003; p for trend 0.004)), higher inflammation (*p* < 0.001; p for trend < 0.001), and a larger proportion had diabetes (p for trend < 0.001) and polypharmacy (p for trend < 0.001), compared to those with highest osteocalcin levels (Q5).

**Conclusion:**

Low osteocalcin in circulation was associated with being frail, also after adjusting for bone turnover markers.

**Supplementary Information:**

The online version contains supplementary material available at 10.1007/s40520-025-03239-6.

## Introduction

The musculoskeletal system has shown to possess more functions besides just mechanical support [1]. The muscle-bone cross-talk encompasses endocrine properties that affect not only the musculoskeletal organ itself, including effects on energy metabolism, but a wide range of other organ systems through a variety of hormones or proteins [1, 2]. In particular, the bone-specific protein **osteocalcin** (bone Gla protein), discovered in the 1970’s [3] and secreted into circulation during bone formation, has gained increasing interest because of its apparent multifunctionality in both humans and animals, counting effects on bone mineralization, energy and glucose metabolism, steroidogenesis, and cognition, at least in mice [1].

Despite this recently recognised multifunctionality, osteocalcin has primarily been studied in humans as a marker of bone turnover in the context of skeletal disorders, such as osteoporosis [4]. We and others have previously investigated osteocalcin in relation to fracture risk and in follow-up after fracture since osteocalcin increases during the fracture healing process [5]. We have also reported that high levels of osteocalcin in urine are associated with an elevated risk of fracture [6].

Others have investigated the association between osteocalcin and muscle function, particularly in response to exercise, although often yielding divergent results. As an example, in human studies increasing levels of serum osteocalcin has been associated with lower muscle strength [7], higher muscle strength [8] or no significant association found [9], making it difficult to draw reliable conclusions. Osteocalcin and it’s different isoforms has also been investigated in relation to other important clinical musculoskeletal outcomes, such as falls [9] and osteosarcopenia [10] but also mortality [11], which all in turn have been linked to frailty. Frailty involves the functional decline of multiple physiological organ systems, where indices quantifying frailty often contain a large musculoskeletal component [12–14].

**Fig. 1:**
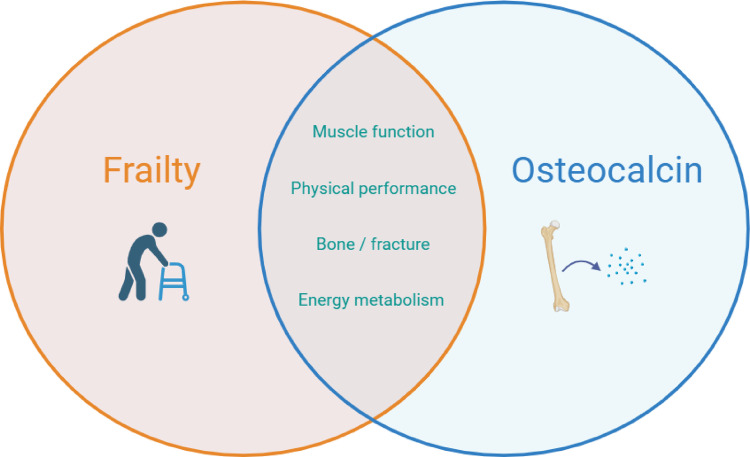
Overlapping outcomes between circulating osteocalcin and frailty syndrome, according to the literature [5, 7, 8, 12, 25, 30, 45]

Given the multiple physiological functions of osteocalcin and the overlapping multi-system nature of frailty (Fig. 1), the aim of the study is to investigate the association between circulating levels of the biomarker osteocalcin and the frailty syndrome. In addition, the study tries to dissect if this assumed association is mediated directly through altered bone turnover or also by other mechanisms.

## Materials and methods

### Subjects

The Osteoporosis Prospective Risk Assessment (OPRA) cohort is a population-based longitudinal cohort of community-dwelling women, all aged 75. The cohort was established to investigate fractures, hence the age criteria for inclusion. In brief, between 1995 and 1999, 1604 randomly selected community-dwelling women from the population files in Malmö, Sweden were invited on their 75th birthday [15, 16]. No exclusion criteria were applied. At baseline 1044 women attended (65% response rate). Reasons for non-attendance included illness (*n* = 152), unwillingness (*n* = 376) or non-response (*n* = 32). Comprehensive evaluation was conducted, including physical performance tests, detailed lifestyle questionnaires and a broad panel of biochemistry analyses. Previous osteoporotic fractures (hip, pelvis, vertebra, distal radius, shoulder) before age 75 were registered [17].

The study was approved by the Regional Ethics Committee in Lund (Dnr: LU200-95 and LU2014804) and performed in accordance with the Helsinki declaration. All participants provided written informed consent.

## Bone turnover markers

Blood samples were collected non-fasting between 8:00 and 13:00, processed within 2 h from phlebotomy and stored at -80˚C. Measurements were done in year 2000, 2001 and 2012 (osteocalcin, CTX and PINP, respectively) using vials not exposed to any prior freeze-thawing and all samples from one timepoint were analysed as a single batch to minimize analytical bias and batch-to-batch variation. **Serum total osteocalcin** (**tOC**), reflecting overall bone turnover, was measured by Elecsys N-MID Osteocalcin immunoassay (S-Total OC; N-MID; Roche Diagnostics, Mannheim, Germany) [16]. Intra- and inter-assay coefficients of variation (CV) were 2,4% and 2,3%, respectively. To test the robustness of our findings, total osteocalcin was also measured by a second assay (in-house, SHOC4) [18]. These two assays of osteocalcin are highly correlated (*r* = 0.90). **Serum intact N-terminal propeptide of type I collagen** (s-**PINP**), reflecting bone formation [19], was detected using IDS-iSYS Intact PINP assay (IDS Ltd, Bolton, UK). Intra- and inter-assay CVs < 4 and < 6%, respectively according to the manufacturer [20]. **Serum C-terminal crosslinked telopeptide of type I collagen** (s-**CTX**), reflecting bone resorption [19], was measured using Elecsys β-CrossLaps ELISA (Roche Diagnostics, Indianapolis, IN, USA). Intra- and inter-assay CVs 5.9% and 5.8%, respectively [16]. Bone turnover markers (BTMs) were analysed either in-house, at the accredited clinical chemistry laboratory, Skåne University Hospital or by Pharmatest Services Ltd in Finland [16, 18, 20].

## Frailty

A frailty index (FI), based on the “accumulation of deficits” model was created [21] according to the principles of Searle et al. [22]. The following variables were included in the FI: muscle strength, average time spent outdoor, gait speed, numbers of steps taken, balance, p-CRP, p-creatinine, mobility/activity level, diabetes, cancer/severe disease, diseases affecting balance, polypharmacy, and self-estimated risk of falling. FI was scored 0.0–1.0, a higher FI indicating higher frailty. An established empirical cut-off of ≥ 0.25 was used to indicate frail status [23]. Complete details of the index are given elsewhere [21]. This index has previously been associated with mortality, falls and fractures [21, 24, 25].

## Musculoskeletal assessments

**Lean muscle mass** (kg) was measured by dual energy X-ray absorptiometry (DXA), as was bone mineral density (BMD, g/cm^2^) (Lunar DPX-L, GE Lunar, Madison, WI). Daily calibration was performed using a phantom. Precision was assessed by duplicate measurements (CV 3.9%, femoral neck BMD; 1.4%, whole-body lean mass). **Muscle strength** was determined using an isokinetic dynamometer (Biodex Medical Systems, v4.5.0, Biodex Corporation, New York) as maximal knee extension (dominant leg), isometric contraction at 90˚ (Nms). **Physical function tests** included balance (modified Romberg test) and gait speed and number of steps taken (2 × 15 m walk with one turn, normal pace) [26–28].

## Statistics

In this study, analyses were performed on the 999 women for whom osteocalcin measurements and frailty index values were available. Descriptive data are presented as mean (SD), median (IQR) or as number of women (%). Levels of total osteocalcin were categorised into quintiles, with comparison made between OC_low_ (Q_1_) vs. OC_high_ (Q_5_). **Association between osteocalcin and frailty** (FI ≥ 0.25) was investigated using logistic regression models (reference category, OC_high_, (Q5)). Potential confounders, determined using Directed Acyclic Graphs (DAG) [29] were included (BMI, vitamin D (s-25(OH)D3), smoking status (yes/no), eGFR (based on plasma cystatin C)). The DAG is included as Suppl. Figure 1. In addition, analyses were performed excluding women on bisphosphonates or glucocorticoids, as these medications supress serum osteocalcin values [30] and/or women with recent fracture (within 2 years, i.e. between age 73–75), as recent fracture increases serum osteocalcin levels [5]. Thus, excluding in total *n* = 135 women.

The different models used were:

**Model 1**: unadjusted with exclusion of bisphosphonate and glucocorticoid users and/or women with recent fracture (women excluded, *n* = 135). **Model 2**: adjusted for BMI, vitamin D, smoking, eGFR, with continued exclusion of bisphosphonate and glucocorticoid users and/or women with recent fracture. **Model 3**: Sensitivity analysis (i), same as model 2 but bisphosphonate and glucocorticoid users and women with recent fracture *included*. **Model 4**: Sensitivity analysis (ii), same as model 2, but without adjusting for eGFR. The reason being that plasma creatinine is included in the frailty index, even though the eGFR in the present study is based on plasma cystatin C. In a final sensitivity **Model 5** (iii), aiming to differentiate the skeletal effects of osteocalcin from other potential effects, we adjusted for the bone markers s-PINP and s-CTX in addition to the adjustments in model 2.

In addition, association between osteocalcin (continuous variable, log-transformed) and frailty index was also investigated using a linear regression model to extend the understanding of this association. Model assumptions were controlled by performing residual diagnostics (scatterplot of standardized residuals vs. predicted values, histogram of residuals, and P-P plot).

To assess potential non-linear association between osteocalcin and frailty, two logistic regression models were applied to data, using frailty status (FI ≥ 0.25) as the dependent variable and adjusted for BMI, vitamin D (s-25(OH)D3), smoking status (yes/no), and eGFR (Cystatin C). The first model included osteocalcin as a linear variable, while the second model incorporated osteocalcin using spline functions with knots placed at the 10th, 50th and 90th percentiles of osteocalcin, as recommended by Harrell [31]. The splines were second order functions between the breakpoints and linear functions at the tails resulting in a smooth curve. The model fit of the two models were compared with likelihood ratio test.

Associations between osteocalcin levels and individual components of the frailty index were investigated using Kruskal-Wallis or Chi^2^ test (p for trend), with post hoc test (Holm-Bonferroni) to counteract false positive findings. Again, excluding women on bisphosphonates or glucocorticoids and/or women with recent fracture, leaving 864 women in the study. As exclusion of these women (*n* = 135) yielded uneven quintiles of osteocalcin, the same calculations were performed with osteocalcin quintiles made *after* exclusions of these women.

## Results

Among the 999 single-aged women in OPRA, serum osteocalcin level ranged from 1.9 to 92.7 ng/ml (median 26.2 (13.9)). General clinical characteristics of the cohort, stratified by quintiles of total osteocalcin, are reported in Table 1. Frailty index (median) from OC_low_ (Q_1_) to OC_high_ (Q_5_) quintile were 0.21, 0.16, 0.15, 0.13 and 0.16, and the frequencies of women who were frail in each quintile were 36%, 22%, 22%, 13% and 23%, respectively.

**Table 1:** Characteristics of the 999 women according to quintiles of serum total osteocalcin at baseline (age 75)

Characteristics	Q_1_ *n* = 199		Q_2_ *n* = 201		Q_3_ *n* = 199		Q_4_ *n* = 201		Q_5_ *n* = 199	
s-total osteocalcin (ng/mL)*	14.8	(4.9)	21.9	(2.3)	26.2	(2.4)	32.7	(3.5)	44.2	(13.0)
Age (years)	75.2	(0.2)	75.2	(0.1)	75.2	(0.1)	75.2	(0.1)	75.2	(0.2)
Weight (kg)*	72.0	(18)	69.0	(13)	66.0	(14)	65.0	(15)	65.0	(15)
Height (cm)	160.9	(5.6)	161.1	(5.3)	160.0	(5.8)	160.9	(5.6)	160.0	(6.0)
Body Mass Index (kg/m^2^)*	27.0	(6.5)	26.5	(5.3)	25.6	(5.0)	25.3	(4.5)	25.1	(5.1)
Frailty index*^a^ Range	0.21 0.05–0.67	(0.16)	0.16 0.05–0.53	(0.13)	0.15 0.02–0.74	(0.13)	0.13 0.01–0.45	(0.10)	0.16 0.01–0.73	(0.13)
s-PINP (ng/mL)*	28.7	(15.7)	41.9	(13.8)	47.1	(16.7)	55.0	(17.5)	74.0	(27.4)
s-CTX (ng/L)*	119.9	(115.4)	225.2	(120.7)	272.9	(145.8)	330.0	(186.1)	510.3	(249.3)
s-25(OH)D3 (nmol/L)*	59	(25)	62	(28)	63	(23)	62	(29)	57	(28)
s-PTH (pmol/L)*	4.0	(2.1)	4.1	(2.1)	3.8	(2.2)	4.3	(2.3)	5.4	(3.1)
p-calcium (mmol/L)*	2.4	(0.1)	2.4	(0.1)	2.4	(0.1)	2.4	(0.1)	2.4	(0.1)
eGFR cysC (mL/min/1.73m^2^)	65	(18)	67	(17)	64	(18)	64	(18)	57	(18)
BMD, femoral neck (g/cm^2^)*	0.797	(0.208)	0.765	(0.189)	0.747	(0.184)	0.740	(0.148)	0.719	(0.177)
T-score, fem. neck (SD)*	− 1.5	(1.7)	−1.8	(1.6)	− 1.9	(1.5)	− 2.0	(1.2)	− 2.2	(1.5)
Lean muscle mass, legs (kg/m^2^)*	4.7	(0.7)	4.7	(0.6)	4.6	(0.7)	4.6	(0.6)	4.7	(0.6)
Total body fat mass (kg/m^2^)*	10.6	(4.2)	10.6	(3.9)	9.9	(4.1)	9.7	(3.3)	9.7	(4.2)

	**No.**	**%**	**No.**	**%**	**No.**	**%**	**No.**	**%**	**No.**	**%**
Recent osteoporotic fracture (age 73–75)	14	(7)	17	(9)	21	(11)	17	(9)	19	(10)
Fallen within the last year	49	(27)	56	(32)	52	(30)	44	(24)	51	(29)
Frail individuals^b^	72	(36)	44	(22)	43	(22)	26	(13)	46	(23)
Current smoker	24	(12)	30	(15)	32	(16)	23	(12)	29	(15)
Alcohol abstainer	46	(23)	27	(14)	36	(19)	40	(20)	44	(22)
Glucocorticoids, current user	11	(6)	7	(4)	5	(3)	2	(1)	3	(2)
Bisphosphonates, current user	22	(11)	5	(3)	2	(1)	0	(0)	1	(1)

*Median (IQR); ^a^Frailty index (cut-off range 0.0–1.0 on each variable); ^b^Frail status (frailty index ≥ 0.25)

s-PINP = Serum intact N-terminal propeptide of type I collagen

s-CTX = Serum C-terminal crosslinked telopeptide of type I collagen

## Low serum osteocalcin is associated with frailty

A logistic regression model shows the association between quintiles of osteocalcin (Q_1_ vs. Q_5_) and frailty status (Table 2). Unadjusted, but excluding women on bisphosphonates or glucocorticoids and/or women with recent fracture (Model 1), the probability of being frail was more than 1.5 times greater among women in the lowest compared to highest quintile of osteocalcin (OR_unadj_ 1.82 [1.12-3.00]). After adjustments (BMI, vitamin D, smoking, eGFR, Model 2), the odds of being frail increased to two and half times in women in OC_low_ (Q_1_) compared to women in OC_high_ (Q_5_) (OR_adj_ 2.55 [1.46–4.44]).

**Table 2:** Logistic regression estimating odds ratio (with 95% CI) for being frail for women in the lowest quintile of osteocalcin (Q_1_), with the highest quintile (Q_5_) as reference (1.0)

	Odds ratio [95% CI]	*p*-value
**Model 1 (unadjusted)** Exclusion of bisphosphonate and glucocorticoid users and/or women with recent fracture	1.82 [1.12-3.00]	**0.015**
**Model 2** Adjusted for BMI, vitamin D, smoking status, and eGFR (CysC). Exclusion of bisphosphonate and glucocorticoid users and/or women with recent fracture	2.55 [1.46–4.44]	**< 0.001**
**Model 3 (sensitivity analysis I)** As for model 2, but bisphosphonate and glucocorticoid users and women with recent fracture *included*	2.76 [1.67–4.56]	**< 0.001**
**Model 4 (sensitivity analysis II)** As for model 2, but without adjusting for eGFR	1.83 [1.09–3.09]	**0.023**
**Model 5 (sensitivity analysis III)** As for model 2, in addition adjusting for s-PINP and s-CTX*	2.50 [1.11–5.61]	**0.027**

*Due to risk of multicollinearity between bone turnover markers (osteocalcin, PINP and CTX), this was tested using correlation matrices and variance inflation factors, which showed acceptable values for including the variables

Bold numbers indicate statistically significant p-values (i.e. *p* < 0.05)

## Sensitivity analyses

Including women on bisphosphonates or glucocorticoids and/or women with recent fracture in sensitivity analysis (i) (Model 3) yielded small effects in the odds for being frail (2.76 [1.67–4.56]). In a second sensitivity analysis (ii) (Model 4), when the analysis was performed without adjustment for eGFR, the odds ratio for women in OC_low_ (Q_1_) was comparable to the unadjusted model (1.83 [1.09–3.09]).

In the final model (iii) (Model 5), adjusted for bone turnover markers s-PINP and s-CTX, the association between low osteocalcin and frailty remained significant (2.50 [1.11–5.61]).

Investigation of association between osteocalcin and frailty using linear regression showed in adjusted analysis, that every 1 SD increase in log-osteocalcin was associated with a -0.021 (95% CI [-0.028-(-0.014)]; *p* < 0.001) change in frailty index (Suppl. Table 1). Osteocalcin explained 4.2% of the variance in the frailty index (partial R^2^ = 0.0416). Residual diagnostics showed no major deviations from model assumptions.

Appliyng logistic regression models to data demonstrated that a non-linear (J-shaped) association between osteocalcin and the probability of being frail was a statistically significant better fit compared to linear model (Suppl. Figure 2).

### Osteocalcin and components of frailty index

The individual components of the frailty index were assessed to determine how strongly they were associated with the quintiles of osteocalcin (Table 3). Characteristic for women in the lowest quintile (OC_low_, Q1) was poorer gait, illustrated by slower gait speed (*p* = 0.001) and more steps needed when walking (*p* = 0.003) compared to women in the highest quintile (OC_high_, Q_5_), whereas muscle strength was similar in the lowest and highest quintile (*p* = 0.484). Women in Q_1_ had a higher degree of inflammation (p-CRP; *p* < 0.001), a larger proportion had diabetes (Q_1_ 18% vs. Q_5_ 3%; p for trend < 0.001) and polypharmacy (Q_1_ 29% vs. Q_5_ 17%; p for trend < 0.001).

**Table 3:** Components of frailty index (continuous or categorical variables as appropriate) in relation to quintiles of osteocalcin

	Osteocalcin						
	Q_1_ *n* = 158	Q_2_ *n* = 175	Q_3_ *n* = 173	Q_4_ *n* = 182	Q_5_ *n* = 176		
s-total osteocalcin (ng/mL)	15.2 (4.6)	22.0 (2.2)	26.2 (2.3)	32.6 (3.4)	44.5 (13.7)		

**Frailty index component**	**Median (IQR)**	**Median (IQR)**	**Median (IQR)**	**Median (IQR)**	**Median (IQR)**	**Uncorrected** **p-value**,** overall** **(p-value**,** Q**_**1**_**vs. Q**_**5**_**)**	**Corrected** **p-value**
Muscle strength – knee extension (Nms)	263 (124)	282 (106)	270 (106)	274 (83)	261 (122)	0.296 (0.484)	0.025
Average time spent outdoor (hours)	2.5 (1.8)	3.0 (1.9)	2.5 (1.9)	2.8 (1.9)	2.5 (1.9)	0.012 (0.158)	0.007
Gait – walking speed (m/s) for 2 × 15 m	1.3 (0.4)	1.4 (0.3)	1.3 (0.3)	1.4 (0.4)	1.4 (0.4)	**< 0.001** (0.001)	0.005
Gait – no. of steps taken, walking 2 × 15 m	49 (9)	47 (6)	47 (9)	47 (8)	47 (9)	**0.004** (0.003)	0.006
Balance (right leg, eyes open) (s)	10 (21)	15 (22)	15 (21)	17 (22)	15 (24)	0.007 (0.011)	0.006
p-CRP (mg/L)	3.0 (4.3)	2.0 (2.6)	1.9 (2.6)	1.5 (2.3)	1.6 (3.0)	**< 0.001** (< 0.001)	0.004
p-creatinine (µmol/L)	74 (16)	73 (16)	74 (19)	73 (17)	77 (20)	0.100 (0.093)	0.013

	**N (%)**	**N (%)**	**N (%)**	**N (%)**	**N (%)**	**p for trend**	
Low mobility/activity level	22 (14)	10 (6)	13 (8)	11 (6)	21 (12)	0.023	0.008
Diabetes	27 (18)	12 (7)	9 (5)	4 (2)	5 (3)	**< 0.001**	0.004
Cancer/severe disease	28 (18)	27 (16)	22 (13)	26 (14)	27 (16)	0.740	0.050
Disease(s) affecting balance, actual	35 (26)	28 (19)	33 (23)	25 (15)	37 (25)	0.133	0.017
Polypharmacy (≥ 5 medications)	46 (29)	28 (16)	29 (17)	20 (11)	29 (17)	**< 0.001**	0.005
High self-estimated risk of falling	19 (14)	16 (11)	9 (6)	9 (6)	13 (8)	0.051	0.010

Bisphosphonate and glucocorticoid users and/or women with recent fracture (fr. 73–75 years of age) excluded (*n* = 135). P-values corrected for multiple comparison using Holm-Bonferroni method

Muscle strength: maximal knee extension, isometric contraction at 90˚ (Nms). Low mobility/activity level: ranging from walking with aid to bedridden (corresponding to cat. 1–4 on a scale from 1–8, with lower number indicating lower mobility level). Diabetes: diabetes at visit (yes/no). Cancer/severe disease: self-reported. Diseases affecting balance: self-reported. High self-estimated risk of falling: self-reported, those with fall risk cat. 4 and 5 on a scale from 1–5 (with higher number indicating higher risk) [21].

Bold numbers indicate statistically significant p-values (i.e. *p* < 0.05) after correcting the raw p-values using the Holm-Bonferroni method

Calculations performed with quintiles of osteocalcin made *after* exclusion of women on bisphosphonates or glucocorticoids and/or women with recent fracture (to ensure even sized quintiles) did not change the results (data not shown).

Finally, in testing the robustness of our data using the second assay of total osteocalcin (SHOC4), results remained similar (data not shown).

## Discussion

This study investigates the association between circulating levels of osteocalcin and the geriatric frailty syndrome among community-dwelling 75-year-old women. In these women, the probability of being frail was J-shaped but with highest probability in women with lowest osteocalcin levels – a finding that withstood adjustment for bone turnover rate i.e. serum markers PINP and CTX.

**Fig. 2:**
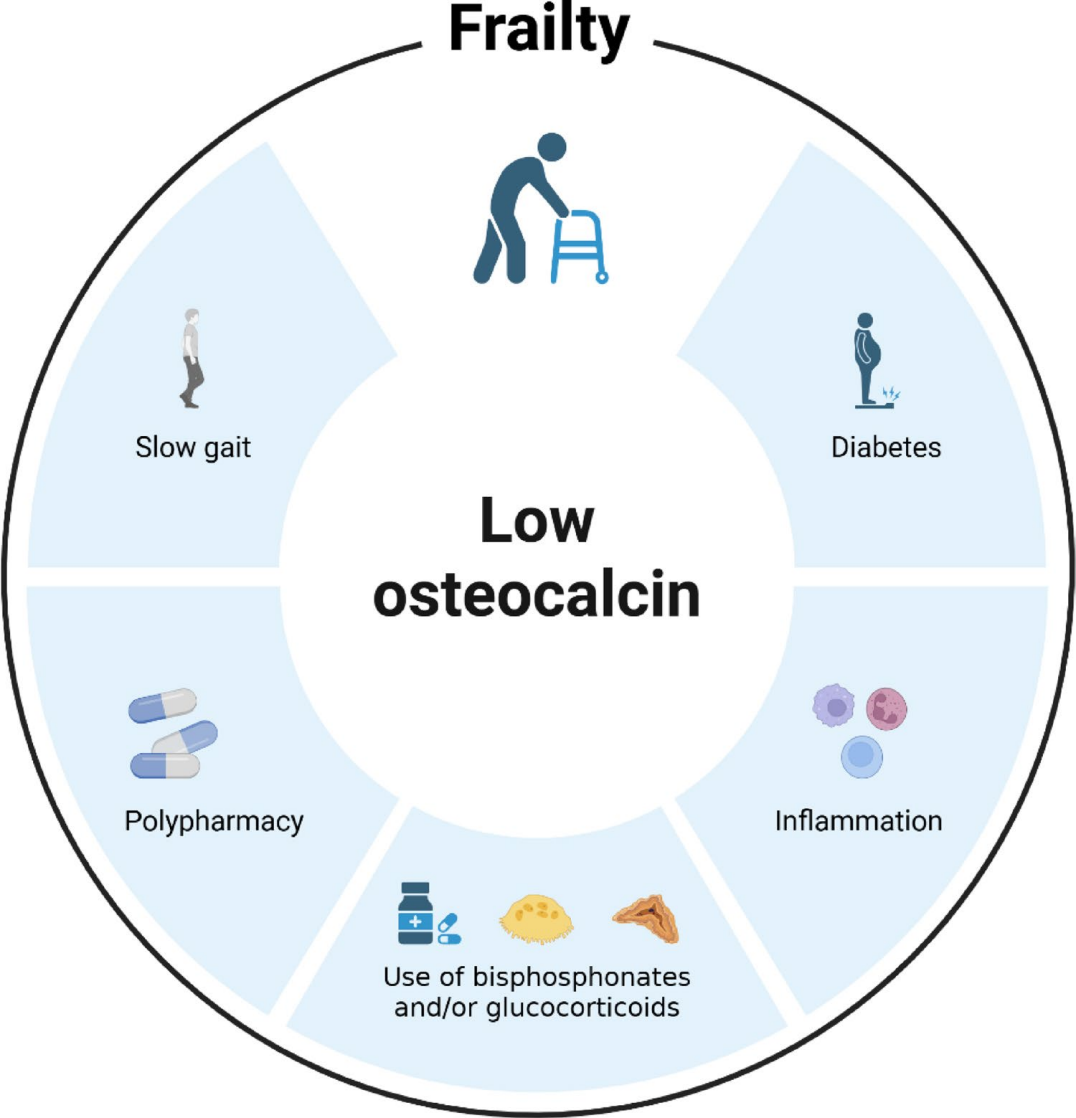
Low osteocalcin is associated with different variables in the frailty index used in this study, graphically illustrated

The frailty syndrome encompasses multiple deficiencies and is associated with adverse consequences such as falls, fracture, and mortality [12, 25]. Correspondingly, osteocalcin has been linked to various organ systems and circulating osteocalcin has been associated with negative outcomes in humans such as falls-related hospitalization [9], muscle function [7, 8, 32], fracture [5, 6, 33], osteoporosis [4], osteosarcopenia [10, 34, 35] and mortality [11, 36, 37]. From this observed overlap, we hypothesized that osteocalcin levels and frailty could be associated. Based on the components of our frailty index, women with the lowest levels of osteocalcin were characterised by poorer gait, worse metabolic status (diabetes), higher inflammation grade and a higher proportion with polypharmacy, in addition to also having higher odds of being frail (Fig. 2).

The association between low osteocalcin and being frail withstood adjustment for specific bone formation and -resorption markers. Osteocalcin is known to be involved in endocrine functions, with poor metabolic status (higher lipid and glucose levels) being associated with lower osteocalcin levels [2, 30]. Women with lowest osteocalcin levels in this study seemed to have higher BMI and total fat mass. In addition, when investigating osteocalcin quintiles and the individual components of the frailty index, there was a significantly higher proportion of women with diabetes in the lowest quintile. Thus, this correlates with previous findings in the literature [30]. In contrast to the expected, given the association between osteocalcin and gait speed, women in the lowest quintile did not have poorer muscle strength. While we cannot fully explain this, other factors possibly contributing are consequences of diabetes, such as polyneuropathy and diabetes-related changes in muscle and bone structure [2, 38]. A greater proportion of these women also had polypharmacy which could indicate higher comorbidity affecting musculoskeletal functioning.

As described above, this study suggests that the association between osteocalcin and frailty is not solely through differences in bone turnover. However, the complexity of osteocalcin precludes definite answers. In general, precautions should be taken when investigating osteocalcin. Apart from different methodologies and isoforms, numerous factors can affect an individual’s osteocalcin level, both in a shorter and longer time perspective [30, 39]. These include age and gender [40], exercise [30], diet [30], medication (e.g. bisphosphonates, glucocorticoids, vitamin K-antagonists) [30], renal function [41], and diseases [2]. In addition, the circadian variation, recent nutritional intake, blood sample handling, and assay applied need to be considered when designing and interpreting studies of osteocalcin [18]. Also, results of animal studies cannot be directly transferred to humans due to differences in gene regulation, protein structure and circadian rhythm of osteocalcin between these species [2]. Nevertheless, in this cohort of older women we found that low osteocalcin is associated with frailty, probably mirroring the interaction between overlapping biophysiological systems. However, the complex interaction is further illustrated by the non-linear association between osteocalcin and frailty which suggests that the probability of being frail was rising again in women with the highest osteocalcin levels, a pattern previously seen in old people and warranting further investigation.

### Strengths

The study has some strengths, firstly the sample size, the cohort is large and population-based with a high coverage, constituting 33% of all 75-year-old-women in the catchment area and with a high participation rate (65%). It is unique as all women were identically aged and of similar ethnicity which reduce confounding [26]. The study was originally constructed to study osteoporosis and fracture; hence, the chosen inclusion age is ideal to also investigate frailty and explore deviations in biological age. In addition, as the perimenopausal period has passed, variation in bone turnover due to hormonal changes is limited. Use of bisphosphonates and glucocorticoids, medications known to influence bone turnover, was in general low (both 3%), and users could therefore be excluded, while keeping a large sample size. Lastly, we have confirmed our results by using two different assays of total osteocalcin, yielding a high correlation (*r* = 0.90) and comparable results, which increases the reliability (data not shown).

### Limitations

The study has some limitations; as the OPRA cohort was not originally designed as a frailty study, the frailty index was developed according to Searle et al. [22], from an initial 40-variable index condensed into a 13-variable index covering relevant physiological domains, and built on accepted principles for constructing a frailty index. Despite this, it correlates well with a larger index from the same cohort, internally evaluated (Spearman’s r^2^ = 0.846) [21]. Samples of BTMs at baseline were collected non-fasting (and as a single measurement), thus, variation in food intake could possibly influence these variables. However, we have previously shown that fasting/non-fasting samples of s-CTX are very highly correlated at later follow-up [6], which probably also applies to the other BTMs since s-CTX is the BTM most sensitive to feeding [42]. Only total osteocalcin concentration was analysed in this study; some details may have been lost due to possible differences in function of the isoforms. Nevertheless, although the endocrine properties of osteocalcin have mostly been related to the under-carboxylated isoform [39], total and under-carboxylated osteocalcin are highly correlated [2]. Despite several adjustments used in the models, we cannot exclude the possibility of residual confounding. We chose to adjust for Cystatin C based eGFR in these older women despite plasma creatinine being included in the frailty index since kidney function might be a substantial confounder in the association between osteocalcin and frailty, and p-creatinine in itself is not an ideal measure in capturing kidney function, especially not in old people. Cognition is an important domain of frailty and known to influence physical function. Likewise, osteocalcin has been inversely associated with cognitive function in mice [43] and in older women [44]. Thus, an estimation of cognitive function would have been valuable, although unfortunately not assessed in this study.

## Conclusion

This study indicates that low levels of osteocalcin are associated with being frail, even after adjustment for bone turnover markers. Frailty is a complex measure to capture loss of health in many organ systems with aging and osteocalcin has known beyond skeletal involvement in many physiological processes; hence, this first study of osteocalcin and frailty generates new questions to be investigated in future studies.

## Supplementary Information

Below is the link to the electronic supplementary material.

Supplementary Material 1

## Data Availability

Data can be made available upon request.
