# Supplementary material for: Osteocalcin and frailty among older women
Source: Aging Clin Exp Res. 2025 Dec 9;37(1):342. doi: 10.1007/s40520-025-03239-6 (PMC12689723; doi:10.1007/s40520-025-03239-6)
Supplement: Supplementary file 1 — Supplementary Material 1 [file 40520_2025_3239_MOESM1_ESM.docx]

**Osteocalcin and Frailty among Older Women**

Tine Kolenda Paulin^1,2^, Linnea Malmgren^1,2^, Patrik Bartosch^1^, Kaisa K Ivaska^3^, Fiona EA McGuigan**^†^**^1^, Kristina E Akesson^1,4^

**Affiliation of authors**

^1^ Clinical and Molecular Osteoporosis Research Unit, Department of Clinical Sciences, Lund University, Malmö, Sweden

^2^ Department of Geriatrics, Skåne University Hospital, Malmö, Sweden

^3^Institute of Biomedicine, University of Turku, 20520 Turku, Finland

^4^ Department of Orthopaedics, Skåne University Hospital, Malmö, Sweden

**Corresponding author**

Tine Kolenda Paulin, MD

Department of Clinical Sciences Malmö, Lund University

Department of Geriatrics, Skåne University Hospital

Clinical Research Centre,

Jan Waldenströms gata 35, 214 28 Malmö, Sweden

Telephone: 040 391131

E-mail: [tine.kolendapaulin@skane.se](mailto:tine.kolendapaulin@skane.se)

ORCID: 0000-0002-7248-2534

**ORCID**

Tine Kolenda Paulin 0000-0002-7248-2534

Linnea Malmgren 0000-0003-3296-7083

Patrik Bartosch 0000-0002-7562-4067

Kaisa Ivaska 0000-0001-7482-7623

Fiona McGuigan 0000-0002-8033-9981

Kristina E Akesson 0000-0003-3024-2804

**
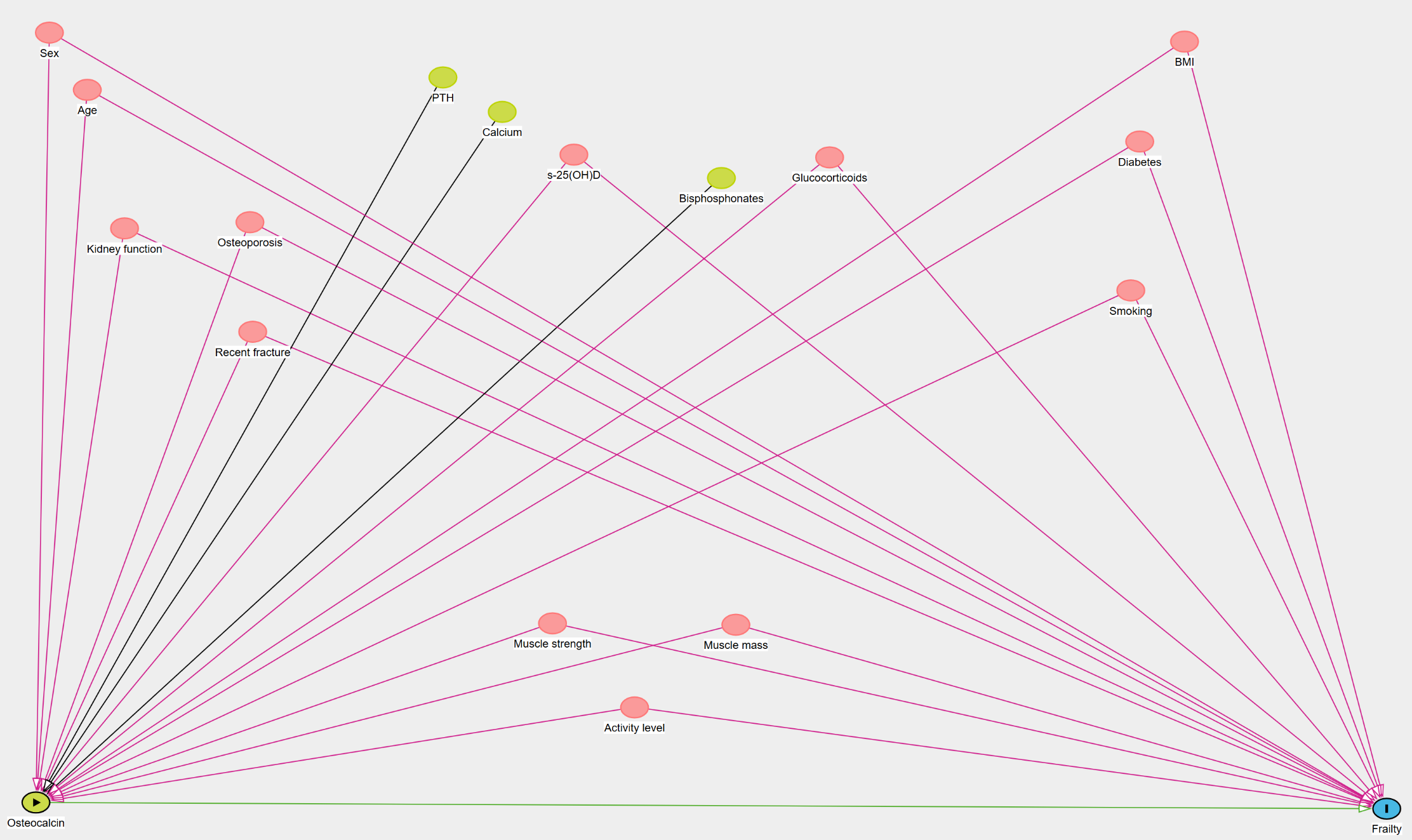
Suppl. Fig. 1**. Directed Acyclic Graph (DAG) that forms basis for selection of confounders and excluded variables in the analyses. Note that some variables might have direct and others indirect (not shown) influence on the exposure and outcome.

**Suppl. Table 1**. Linear regression models estimating the association between log-standardized osteocalcin (per-SD increase) and frailty index.

| **Model** | **β (per 1-SD increase in osteocalcin)** | **95% CI** | **p-value** | **Partial R^2^**  **(osteocalcin)** | **Model R^2^** |
| --- | --- | --- | --- | --- | --- |
| Model 1.  Unadjusted* | -0.012 | -0.020-(-0.005) | **<0.001** | 0.0128 | 0.0128 |
| Model 2.  Adjusted for BMI, vitamin D, smoking status, eGFR (Cys C)* | -0.021 | -0.028-(-0.014) | **<0.001** | 0.0416 | 0.1384 |
| Model 3 (Sensitivity analysis I)  As for model 2, but bisphosphonate and glucocorticoid users and recent fracture *included* | -0.023 | -0.029-(-0.016) | **<0.001** | 0.0484 | 0.1406 |
| Model 4 (Sensitivity analysis II)  As for model 2, but without adjusting for eGFR* | -0.013 | -0.020-(-0.006) | **<0.001** | 0.0151 | 0.0497 |
| Model 5 (Sensitivity analysis III)  As for model 2, in addition adjusting for s-PINP and s-CTX* | -0.016 | -0.028-(-0.005) | **0.005** | 0.0098 | 0.1429 |

*Exclusion of glucocorticoid and bisphosphonate users and recent fracture (from 73-75 years of age).

Bold numbers indicate statistically significant p-values (i.e. p<0.05).

**Suppl. Fig. 2.** Two logistic regression models, spline (red line, 95% CI dashed) vs linear model (black line, 95% CI dashed) of the adjusted association between osteocalcin and frailty probability. Knots at 10th, 50th, and 90th percentiles (15.6, 26.5, 45.2 µg/L). Both models adjusted for BMI, vitamin D, smoking, and eGFR (Cys C). Spline model fit significantly better (p<0.001) and indicated a J-shaped association.
